# Application of Catalysts Prepared by Solution Combustion Synthesis in Dry Reforming of Methane

**DOI:** 10.3390/molecules30234575

**Published:** 2025-11-27

**Authors:** Svetlana A. Tungatarova, Alua M. Manabayeva, Arlan Z. Abilmagzhanov, Tolkyn S. Baizhumanova, Makpal K. Malgazhdarova

**Affiliations:** D.V. Sokolsky Institute of Fuel, Catalysis and Electrochemistry, 050010 Almaty, Kazakhstan; manabaeva_2018@mail.ru (A.M.M.); a.abilmagzhanov@ifce.kz (A.Z.A.); baizhuma@mail.ru (T.S.B.); m.malgazhdarova@ifce.kz (M.K.M.)

**Keywords:** methane, dry reforming, catalyst, solution combustion synthesis, synthesis gas, hydrogen

## Abstract

Dry reforming of methane (DRM) is a method whereby two greenhouse gases (methane and carbon dioxide) are synthesized into a high-value gas. Suitable catalysts with optimal compositions are still in development, as problems concerning coking and metal sintering remain unresolved. Since the late 20th century, catalysts prepared via solution combustion synthesis (SCS) have been applied for catalytic reactions, as these materials (catalyst or supports) demonstrate high catalytic performance; for example, SCS catalysts have been tested in DRM. This review describes the history of solution combustion synthesis, compares it with traditional methods of preparing catalysts for DRM, and charts recent developments in SCS catalytic systems based on Ni and Co. SCS catalysts are prepared by burning nitrates (oxidizing agents) and fuels (reducing agents) at mild pre-ignition temperatures. In this review, the effects of fuel type and mixed-fuel systems on the catalyst composition, as well as its activity in DRM, are described. These catalysts have shown high metal dispersion, good coke resistance, and stable catalytic performance in long-term tests. This review demonstrates the main reasons for catalyst deactivation, such as coke deposition on the catalyst surface, and suggests ways to reduce them.

## 1. Introduction

Global oil reserves are diminishing daily due to continuous production and processing using cutting-edge technologies [1]. Around the world, scientists are exploring various feedstocks and ways to utilize the vast natural gas resources as a substitute for oil. Significant attention has been paid to natural and associated gases as an alternative feedstock source for the petrochemical industry [2].

Prospects for the development of the global gas-processing industry are linked to the development and implementation of new, clean catalytic technologies for producing syngas based on fuel blends. In recent years, natural gas has received increasing attention as a potential feedstock for the chemical industry. The first step in natural gas conversion is often the targeted production of syngas as a clean, modern fuel [3]. Syngas can then be further used in various petrochemical syntheses (e.g., Fischer–Tropsch) to produce valuable chemical products [4]. The rational utilization of natural and associated petroleum gases and the elimination of their flaring are two of the most pressing and unresolved environmental issues at present. In the context of the crisis and the sharp decline in natural resource prices, both natural and associated petroleum gases can be considered alternative sources of valuable petrochemicals and organic synthesis products, which are highly prized on the global market.

Syngas is produced from DRM, offering a means of carbon capture, utilization, and storage. Recent reviews, regardless of the utilization of CO_2_ emissions through methane reforming processes, have revealed that in the case of hydrogen production, the global output from steam reforming exceeds 60 million tons annually. However, ca. 30 million tons of CO_2_ emissions could be eliminated each year through DRM, effectively creating a significant anthropogenic carbon sink equivalent to approximately 0.25 gigatons per year in the current CO_2_ market. Since CH_4_ and CO_2_ atoms both contain carbon atoms, the main issue associated with DRM is carbon deposition on the catalyst. High temperatures usually cause metal sintering. Researchers hope that integrating DRM into the industry will ameliorate global climate change, improve syngas production, and help maintain carbon neutrality [5].

Since this reaction was discovered, many catalysts have been investigated for use in DRM [5,6], some of which are noble metals. However, due to their high cost and unavailability, noble metals have been replaced by nickel and cobalt. Ni catalysts in particular suffer from metal sintering and coke deposition, although they demonstrate high catalytic performance. Ni and Co catalysts can be improved by selecting suitable preparation methods, pretreatment conditions, promoters, supports, and DRM reaction conditions.

One such method of preparation is SCS, which has been in development for the last 20 years. Initially, SCS materials were used in nanotechnology, space ships, and applications requiring high-temperature resistance [7]. SCS later attracted interest for its potential use in catalytic reactions [8,9,10,11,12] due to its temperature stability and resistance to metal sintering, i.e., desirable characteristics for DRM, in which reaction temperatures are high [13]. SCS materials are highly dispersed, offering good catalytic performance during DRM [14,15,16].

The aim of this work is to provide information on different preparation methods, focusing on SCS—its advantages, disadvantages, and application for the preparation of catalysts for DRM—along with the reasons for catalytic deactivation and means of decreasing carbon deposition. This article offers a detailed discussion and thorough analyses and presents the future prospects of DRM and its catalysts.

## 2. Solution Combustion Synthesis as Method of Preparing Catalysts for Dry Reforming of Methane

In this section, oxide catalysts and materials prepared via self-propagating high-temperature synthesis (SHS) and solution combustion synthesis (SCS) are described. The latter materials can be tested in DRM and other catalytic reactions due to their thermal stability, high dispersion, and good reducibility.

### 2.1. History of Solution Combustion Synthesis

SHS is a new type of combustion in which solid materials are the final products. It was first developed in 1967. Self-propagating high-temperature synthesis is an exothermic process that occurs very quickly due to its self-sustaining chemical reactions. SHS offers significant advantages over other methods, namely, its simplicity and low cost. A distinctive feature of this process is that the heat required for the reaction to occur is provided by the reaction itself. However, the ignition temperature must be reached to initiate the process. SHS is used to produce a variety of refractory inorganic compounds, intermetallics, catalysts, and metallurgical powders. The resulting products are of high quality and require no additional processing [17].

Since the discovery of SHS reactions, great strides have been made, and alternative SHS technologies have been developed, such as sol–gel and high-energy milling [18,19]. Most of these methods require multiple synthesis steps, specialized equipment, and energy-intensive measures. For example, a disadvantages of the sol–gel method include the instability of the sol over time and its sensitivity to temperature fluctuations, typically addressed by introducing various additives, which hinder the interaction of particles with the solvent.

To overcome these shortcomings, a technically simpler single-stage process called “solution combustion” was developed in the mid-1980s. This new, modified SHS method was proposed in 1988 by Indian scientists [20]. Finely dispersed α-Al_2_O_3_, β-Al_2_O_3_, and aluminum-containing spinels, such as MgAl_2_O_4_, CaAl_2_O_4_, LaAlO_3_, and Y_3_Al_5_O_12_, were synthesized by this method. Metal nitrates were applied as oxidizing agents, and urea was used as a reducing agent. It was found that during a redox reaction, the mixture of initial reagents ignites, resulting in the formation of composite materials. This method is used to produce metal oxides and is applied in various other industrial fields. Due to the widespread use of this method, the materials’ composition, structure, and properties have been systematically studied.

At the Institute of Nanoscience and Nanotechnology, Demokritos National Center for Scientific Research (Greece), under the supervision of Xanthopoulou, studies are being conducted on the microstructure and properties of synthesized composite materials, the mechanisms of physicochemical synthesis, and the dynamics of product formation [21].

In Japan, thermites applied for the manufacture of components and products for use in aerospace engineering and space exploration were prepared using SHS technology by Odawara [22], who achieved success by controlling processing parameters, such as microwave launch (Equation (1)):

(1) $$M^{\nu }{\left({NO}_{3}\right)}_{\nu }+\left(\frac{5}{9}\nu \varphi \right)C_{2}H_{5}{NO}_{2}+\frac{5}{4}\nu (\varphi -1)O_{2}\to M^{\nu }O_{\frac{\nu }{2}(s)}+\left(\frac{10}{9}\nu \varphi \right){CO}_{2(g)}+\frac{25}{18}\nu \varphi H_{2}O_{\left(g\right)}+\nu (\frac{5}{9}\varphi +1)/2N_{2(g)}$$

where ν is the valence of the metal. If φ = 1, the initial mixture does not require atmospheric oxygen for complete fuel oxidation. If φ > 1, excess fuel is present. If φ < 1, then there is insufficient oxygen for combustion [23]. Solution combustion synthesis is primarily used to produce porous ceramic or metallic materials. This process is characterized by moderate furnace heating temperatures (350–600 °C). Products obtained by this method typically have high purity and the ability to crystallize with nanoscale material clusters.

The authors of [24] noted that combustion synthesis can proceed in two main modes: SHS and volumetric combustion. The SHS mode enables foil formation during combustion, while bulk combustion processes—such as reactive sintering, reactive welding, and spark plasma sintering—show promise for obtaining materials that are difficult or even impossible to produce by conventional methods. These approaches have been successfully applied to the synthesis of dissimilar systems, including ceramics, metals, and refractory materials such as graphite, carbon–carbon composites, and W-, Ta-, and Nb-based compounds. Building on this, recent advances and trends in combustion science toward nanomaterial synthesis are discussed in [25], where various modifications to traditional combustion methods are critically analyzed. Special attention is paid to the preparation and potential applications of nanoscale products obtained through combustion synthesis.

The authors of [26] report new approaches and results achieved in the synthesis of materials through solution combustion. SHS and SCS methods were developed by Manukyan and Mukasyan [23,24]. Mukasyan also collaborated with the Indian scientist Varma. They discussed the thermodynamic and kinetic principles of self-propagating combustion reactions and the principles for controlling the composition and structure of composite materials [26]. The latest systems developed for the formation of new materials and unique structures for producing thin films and two-dimensional crystals with unusual properties are described. The synthesized materials are used in catalysis, for the preparation of optical devices and batteries, and in various nanoceramics, including in bio-, electrical-, and magnetic devices. In the field of porous material synthesis, solution combustion enables the growth of thin metal oxide films at low temperatures, facilitating the production of inexpensive and high-performing electronics on flexible plastic substrates [27].

SCS catalysts have been widely applied in heterogeneous catalysis for exhaust emission control, hydrogenation reactions, hydrogen production, and photocatalytic processes, including oxidation reactions [28].

Thus, SCS offers several advantages, such as rapid synthesis, low energy consumption, inexpensive reagents, and the ability to form thermally stable mixed oxides and spinels, catalysts that can be applied in heterogeneous catalysis, environmental remediation, and hydrogen production.

### 2.2. Dry Reforming of Methane for Synthesis Gas Production

In DRM, an oxidizing agent, i.e., CO_2_, oxidizes CH_4_ to CO and H_2_. Hydrogen is the clean fuel of the future because burning it does not cause greenhouse gas emissions. Hydrogen production technologies using renewable resources are beginning to develop to ensure a sustainable energy cycle (Equation (2)) [29].

CH_4_ (g) + CO_2_ (g) → 2CO (g) + 2H_2_ (g) (2)

During DRM, a parallel reverse water–gas shift reaction (Equation (3)) occurs, increasing CO_2_ conversion and decreasing the yield of H_2_, which reduces the H_2_/CO ratio to below unity [30,31].

The catalytic POM has garnered significant interest due to its ability to produce a favorable H_2_/CO ratio equal to 2.0, which is ideal for methanol manufacture and the Fischer–Tropsch reaction.

H_2_O + CO ↔ CO_2_ + H_2_ (3)

DRM is important to industry and suitable for producing syngas with an equimolar ratio of H_2_ and CO [32,33,34]. This syngas composition is particularly advantageous for applications like Fischer–Tropsch synthesis. Furthermore, the DRM reaction is environmentally friendly and aligned with the principles of green chemistry, as it transforms greenhouse gases (specifically, CH_4_ and CO_2_) into valuable feedstock or intermediate products for use in industry [35]. This reaction requires robust basic catalysts, and many studies have been dedicated to developing catalysts that demonstrate high performance and stability. Supported metal catalysts (inexpensive and available Ni and Co) are commonly employed in hydrocarbon reforming and are typically prepared through impregnation of various supports [36]. Notably, full reproducibility is difficult to achieve with this method, as metal can be distributed irregularly on the surface of the catalyst, leading to inconsistencies. Additionally, the fine metal particles obtained through this process tend to sinter at elevated temperatures, ultimately deactivating the catalyst. It is widely acknowledged that most group VIII metals display selectivity to syngas and optimal conversion (CH_4_ and CO_2_) [37].

Many catalytic systems that show high activity in DRM have also been explored in SRM, providing valuable insights into the structure–activity relationships relevant to this study. In particular, Ni-based mixed oxides, such as NiMgAl and NiCeAl, showed high performance in both reactions due to their enhanced dispersion, resistance to sintering, and improved metal–support interactions. These comparative observations are noted only to highlight catalyst design principles that are also applicable to DRM.

The difference between catalysts for two endothermic methane-reforming processes lies in the use of different oxidants, i.e., CO_2_ (DRM) and steam (SRM). As a result of this difference, the reaction conditions vary from process to process. The catalytic properties depend on the type of oxidant used; thus, oxidants with specific properties are selected. For instance, steam is a strong oxidant due to the lower dissociation energy of its O-H bonds (ca. 460 kJ·mol^−1^) compared to the bond energy of C=O (ca. 805 kJ·mol^−1^). Alumina can be a good support for both DRM and SRM reactions, such as in the well-known Ni/Al_2_O_3_, although this catalyst is prone to coke deposition due to its acidity. DRM catalysts require supports and promoters with moderate basicity and acidity, as well as redox properties (oxygen storage capacity) to activate inert CO_2_ molecules. MgO, MgAl_2_O_4_, CeO_2_ (La_2_O_3_), CeO_2_-Al_2_O_3_ (La_2_O_3_-Al_2_O_3_), and alkaline metals are good promoters and/or supports for DRM because they offer strong metal–support interactions, which are important for this process [38]. On the contrary, in SRM, supports should not react with the steam, as such a reaction causes hydroxylation of oxide surfaces, damaging the catalyst. Sintering and catalyst damage are disadvantages of SRM in terms of catalyst performance [39]. Regarding CO_2_ emissions and energy consumption, SRM is less favorable because it emits CO_2_ and necessitates a water evaporator, which is an additional expense. It is noteworthy that DRM consumes CO_2_.

The difference between these processes lies in their catalyst requirements. In steam reforming of methane, catalysts depend on metallic nickel or noble metals to enable the reaction with steam. In contrast, in dry reforming of methane, catalysts typically comprise combinations of metals, such as nickel and cobalt, or are designed with specific supports that enhance resistance to carbon deposition and maintain activity toward both methane and CO_2_. Metallic nickel is essential for catalytic activity in SRM, but DRM catalysts must be carefully structured to minimize carbon buildup, which is a major challenge associated with this reaction.

Detailed descriptions of the optimal catalysts, active components, promoters, and carriers used in DRM, its advantages and disadvantages, and the effect of varying the reaction mixture, flow rate, and duration of the experiment are provided in other sections.

### 2.3. Method of Preparing Catalysts

Metal dispersion and surface area can be enhanced by selecting a suitable method of preparing supports. Incorporation of active metal into support or building core–shell structures to prohibit metal sintering in DRM is currently under study.

How a catalyst was obtained substantially influences its physicochemical features and functionality. Two universal classical approaches to producing catalysts are co-precipitation and impregnation. When comparing the sol–gel approach to these conventional approaches, the sol–gel method can produce a catalyst with certain advantages, such as excellent purity, a fine size placement, a slower deactivation rate, and excellent thermal counteraction contrary to agglomeration [40].

Important criteria for catalysts are high dispersion and a narrow size distribution on the support, which can be achieved through impregnation [41,42,43]. The advantage of impregnation lies in its large batch capacity and high efficiency. Most of the active component is distributed on the surface of the support and is effectively utilized. The main drawback of this method is that it is difficult to control the uniform distribution of active components on the support, which affects the final particle size and their thermal stability. However, problems relating to non-uniform distribution, weak metal–support interactions, and sintering at high temperatures can be solved via multiple immersions in a diluted solution [44].

Research on the synthesis of inorganic nanomaterials via the hydrothermal method [45] began in the nineteenth century, and another century passed before studies in the field of functional materials were initiated. Simply put, the hydrothermal method involves the use of a high-temperature and high-pressure medium in a reactor to dissolve substances that are difficult to dissolve under normal temperature and pressure conditions. After the reaction, crystals precipitate, and crystal growth is controlled by adjusting the reaction time to obtain the desired grain size. The advantage of the hydrothermal method is that the synthesized product exhibits good and controllable crystallinity, and its disadvantage is the high cost of the reaction equipment.

The sol–gel method is a process of forming oxides or other solid compounds from organic or inorganic metal compounds through sol–gel formation, followed by heat treatment [46]. Inorganic or organometallic substances can be used as precursors to obtain sols. The precursor and solvent react to form a gel with a three-dimensional network structure, and a nanopowder is prepared through subsequent stages of drying and sintering. The advantage of the sol–gel method is that it ensures uniform mixing at the molecular level at a relatively low reaction temperature. The disadvantages are the long reaction time and the high porosity of the gel, which decreases significantly during drying, causing considerable material shrinkage.

The microemulsion technique involves mixing immiscible liquids to form nanosized droplets [47,48,49,50,51,52,53]. Since the droplet size in a microemulsion is on the nanometer scale, a nanoparticle precursor containing the active component is added to the microemulsion; the droplets can then be used to control the nanoparticle size, achieving atomic-level mixing. Catalyst nanoparticles obtained by microemulsion have uniform size and good dispersion. To prevent the sintering of metal nanoparticles during catalysis, the metal particles must be tightly anchored to the support. The calcination temperature affects the mean metal particle size: the higher the calcination temperature, the higher the mean metal particle size [40,53]. According to the BET method, the surface area of Ni@SiO_2_ calcined at 550 °C is equal to 112 m^2^·g^−1^; for Ni/SiO_2_–650, this value is increased to 203 m^2^·g^−1^. Thereafter, the surface area is reduced when the treating temperature increases. Methane and CO_2_ rates decreased as the calcination temperature increased from 750 °C to 950 °C. These temperatures strengthen catalysts during stability tests, making them more durable, which correlates with strong metal–support interactions (MSI).

Pretreatment involving calcination modifies the structure of the support materials. Lower calcination temperatures can lead to partial disintegration of the metal precursor, thus decreasing its content in the active composite. Conversely, higher temperatures can induce sintering of these active composites, leading to a decline in deformation of the support structure and surface area. For example, 5 wt.% Ni/ZrO_2_ was produced by impregnation and underwent calcination at 600 °C, 700 °C, and 800 °C. The sample calcined at 800 °C exhibited high conversion (CH_4_–75%, CO_2_–60%). It was noted that surface area decreased at higher temperatures [54].

Table 1 provides a comparison of SCS catalysts prepared using different methods. Synthesized catalysts showed notable differences in structure, textural properties, and performance in DRM (Table 1). Catalysts prepared by the SCS method, such as Ni–La_2_O_3_ and Mg–Ni–La_2_O_3_, exhibit moderate surface areas (37–54 m^2^ g^−1^) and stable conversions of up to 80–90% for both CH_4_ and CO_2_ at 700 °C, with H_2_/CO ratios near unity [55]. The PACS and sol–gel routes generally yield higher surface areas (up to 184 m^2^ g^−1^) and enhanced initial conversions of 90–97%, maintaining stability over time [56,57]. In contrast, impregnated catalysts like Ni–Al_2_O_3_–CeO_2_ [44] and Ni–La_2_O_3_ [58] perform moderately well (60–85% conversion) and are more prone to deactivation. Co-precipitated systems, for example Ni–Mg/Al_2_O_3_ [59] and Ni–Fe/Mg(Al)O [60], achieve conversions of 90–99% with high stability even after 200 h of operation, owing to strong metal–support interactions and basicity. Perovskite-type oxides such as LaNi_0.9_Ru_0.1_O_3_ [61] and La_0.8_Sr_0.2_Ni_0.8_Fe_0.2_O_3_ [62] exhibit improved thermal stability but require higher temperatures for activation. Catalysts prepared by different methods exhibit different physico-chemical characteristics and performances in DRM. The main criteria for selecting an optimal catalyst are the calcination temperature and the chemical composition of the catalyst [63,64,65,66,67,68,69,70,71,72,73,74,75,76,77,78,79,80,81,82]. Overall, SCS and co-precipitation methods provide the best combination of dispersion, surface area, and long-term stability, while impregnation and sol–gel catalysts show relatively faster deactivation or lower methane activation efficiency.

Hence, widely applied methods, i.e., impregnation and co-precipitation, are simple and scalable but associated with disadvantages in controlling the uniformity of metal distribution, thermal stability, and metal–support interactions. Microemulsion and sol–gel methods are more advanced and offer superb control of particle size, with stirring at the molecular level. Nevertheless, these methods are often more expensive, necessitate more preparation, or produce catalysts that suffer from structural shrinkage during drying.

A comparative analysis of catalysts prepared by distinct methods demonstrated that SCS and co-precipitation generally offer the most suitable balance between reducibility, metal dispersion, resistance to deactivation, and textural properties. SCS-generated catalysts show high stability and near-equilibrium conversions, while co-precipitated systems exhibit good long-term performance due to robust metal–support interactions. The sol–gel and PACS methods yield large surface areas and high activity, although they are susceptible to faster deactivation. Impregnated catalysts offer moderate performance with a tendency toward metal sintering. Overall, careful selection of the synthesis method is essential for optimizing catalyst performance, durability, and resistance to coke deposition in DRM.

### 2.4. SCS Catalysts for Dry Reforming of Methane

Fewer studies have investigated SCS as a means of obtaining catalysts. In this approach, a solution containing nitrates and fuel undergoes combustion at 300–600 °C. Therefore, certain materials, for instance, luminescent nanospheres, quasi-conductors, bioceramics, and unique nano-oxides, can be manufactured via SCS [23]. Catalysts obtained through this process can be effective in DRM because of their narrow particle size, which increases catalytic activity [83].

SCS can produce supported and bulk catalysts. Catalysts of the first class are froth-like materials applied in numerous catalytic reactions. Pristine metals, oxides, and alloys can be prepared. For example, LaFeO_3_ perovskites can be used in autothermal reforming of hydrocarbons and perform well. Catalysts of the second class can be obtained at high temperatures via precipitation of active metals onto the support. In [84,85], a pre-ignition temperature of 300–600 °C was observed to initiate fast elevated-temperature combustion. Water evaporation and gel formation occurred in a few seconds during the first stage of burning. Thereafter, gel combustion in the air medium occurred with a blaze, forming highly dispersed nickel in the form of 5 nm particles (Equation (4)). The reaction of fuel with nitrates proceeds differently depending on the fuel used. Combustion gases (CO_x_ and NO_x_) and heat are released during micro-explosions. Notably, the flame temperature is higher than the pre-ignition temperature. Solution burning cannot be initiated if the pre-ignition temperature is below 300 °C. A higher temperature decreases the explosion time and enhances the crystallinity of the resulting catalyst [26].

An exothermic reaction of urea and Al(NO_3_)_3_·9H_2_O is demonstrated further (Equation (4)):

2Al(NO_3_)_3_·9H_2_O +5CH_4_N_2_O = Al_2_O_3_ + 8N_2_ + 5CO_2_ + 28H_2_O (4)

Ultimately, only metals with different oxide forms, for instance, oxide, alloy, perovskite, and spinel, remain.

This method of preparation is called the synthesis of catalysts by the combustion of a solution, or solution combustion synthesis. When a gel is obtained by thoroughly stirring fuel and nitrate, followed by placing this mixture into a furnace, it is called the combustion method. In other cases, fuel and nitrate are mixed in water at 90 °C on a heater, and the obtained gel is burnt at temperatures of 500 °C or higher.

Studies [86,87,88] report that nickel aluminate (NiAl_2_O_4_) is formed during solution combustion synthesis (SCS), particularly at elevated pre-ignition or calcination temperatures between 500 °C and 900 °C. XRD analyses confirmed the coexistence of NiO and NiAl_2_O_4_ phases and the absence of alumina, indicating complete incorporation of aluminum into the spinel structure. It was further demonstrated that high-temperature calcination above 800 °C promotes the crystallization of a stable NiAl_2_O_4_ bulk phase in which nickel is uniformly integrated. Subsequent reduction of this spinel leads to the formation of Ni/Al_2_O_3_ catalysts with finely dispersed Ni particles (10–30 nm), providing enhanced activity at moderate reaction temperatures and strong resistance to coking and particle sintering.

Table 1 contrasts between various catalyst preparation methods, in which all catalysts are reduced prior to the reaction.

The findings affirm that adding Mg does not improve the dispersion of nickel within the La_2_O_3_ pattern (Table 1). Instead, the size of the Ni particle increased with the addition of the modifier. This contrasts with Ni–La_2_O_3_, which is attributed to a weakening of MSI caused by the introduction of the promoter. The use of both Ni–La_2_O_3_ and Mg-Ni–La_2_O_3_ resulted in a slow increase in conversion with time on a gas stream [55]. The partial reduction of nickel oxide species throughout the pre-treatment process induced an increase in activity over time. This improvement, observed in the initial conduction period, is linked to gradual progress toward the La_2_O_2_CO_3_ equilibrium concentration. Lanthanum dioxycarbonate is formed by the carbonation of La_2_O_3_. Once a steady state was reached, conversion remained constant for 100 h of operation, while a decline in activity was observed in conversion of Ni–La_2_O_3_ and Li–Ni–La_2_O_3_. The fact that a reverse water–gas shift (RWGS) reaction occurred can explain the previous statement. Mg is observed to improve catalytic activity [57]. The use of an Al_2_O_3_-CeO_2_ support [44] in a Ni-containing catalyst also tends to strengthen catalytic performance; however, there is less CH_4_ conversion than when Ni/Mg-Al is used. For the above-mentioned catalysts, the conversion of CO_2_ was higher than the conversion of CH_4_; therefore, the H_2_/CO ratio was close to unity during DRM. The catalyst containing the NiOMgO solid solution was prepared by a modified SCS method called paper-assisted combustion synthesis (PACS), in which cellulose paper and glycine are used as fuel [56]. With this cellulose sheet, material was dried at 80 °C for 2 h and then ignited at 200 °C within a few min. It is worth noting that this method does not require high temperatures to synthesize catalysts. In comparison with SCS, the modified variant, PACS, improved the catalytic activity of the catalysts, as shown in Table 1. Another modified SCS method is colloidal combustion synthesis (CCS), in which active metals, promoters, colloidal support (SiO_2_), and glycine dissolved in deionized water are ignited together. Combustion is induced with an oxidant (metal nitrates) and fuel (glycine) at a pre-ignition temperature of 230 °C. Then, calcination occurs at 600 °C for 4 h. The disadvantage of this method compared with PACS lies in its lower CH_4_ conversion (77%), high calcination temperature, and long duration of calcination [56].

Thus, the combination of a novel catalytic material and preparation method would improve the long-term performance of the catalyst in dry reforming.

It is obvious that wet impregnation requires many steps during preparation, while for SCS, two steps are enough to obtain an efficient, stable, and well-dispersed catalyst with a low surface area. SCS is in no way inferior to impregnation; on the contrary, it offers a number of advantages [63].

In colloidal combustion synthesis, colloidal SiO_2_, glycine, and a metal nitrate (for example, Ce(NO_3_)_3_) are ignited at 150 °C. As a result, ceria nanoparticles are formed between SiO_2_ colloids. CeO_2_ prepared with this method can have large pores (ca. 20–22 nm) and a large surface area, depending on the amount of silica used [89]. If the silica volume is increased, the surface area is improved. The ignition temperature also depends on the silica volume. The higher the amount of silica, the lower the ignition temperature is. When the amount of silica is too high, ignition cannot occur.

Overall, SCS is a highly promising method of preparing catalysts with finely controlled structural and textural properties. Spinel, oxide, perovskite, and alloy phases with high dispersion can be obtained with this method. The intense, rapid combustion results in the formation of nanosized active phases at comparatively low pre-ignition temperatures (300–600 °C). These phases yield finely dispersed Ni particles with robust metal–support interactions and pronounced resistance to coking and sintering, enhancing catalytic performance in DRM. Hence, SCS is a powerful strategy for developing durable and efficient catalysts for dry reforming of methane.

### 2.5. Effect of Fuel Content on Solution Combustion

To understand the mechanism of solution combustion, it is necessary to elucidate the role of organic fuel in the initial catalyst composition. The fuel-to-oxidizer ratio, as well as the amount of water in the solution, significantly influence the thermodynamic parameters, including enthalpy, adiabatic combustion temperature, and total amount of decomposition gas. Fuel types are interrelated with powder characteristics, such as the crystalline structure, particle size and shape, and specific surface area of the resulting powder.

Typically, successful combustion synthesis is not vigorous, resulting in the release of non-toxic gases and excellent complexing properties between metal salts and fuel. For example, when heating a solution, water must be evaporated, which affects the stability of the metal ions in the solution. Therefore, the coordination capacity of the fuel, the type of metal salt, and the heating rate are key factors in the synthesis of target materials. The type and amount of fuel and metal salts also determine the combustion temperature and the amount of gases released [90].

Various organic fuels are used as reducing agents in solution combustion. These can include urea, glycine, aminobenzoic acid, hydrazine, citric acid, sucrose, glucose, starch, and other organic substances. All of these fuels serve two primary purposes:

- They are sources of C and H which, when burned, form CO_2_ and H_2_O and release heat;
- They form complexes with metal ions and provide a source of nitrogen (during solution combustion, CO and NH_3_ are formed, which are reactants necessary for obtaining metals in the solution).

The type of fuel and the fuel/oxidizer (F/O) ratio directly affects the flame temperature (the exothermicity of the solution combustion reaction) [91]. Fuel choice is made based on the enthalpies of the oxidizer and the fuel. For example, ΔH_f_ of urea is −333.5 kJ·mol^−1^, and ΔH_f_ of glycine is −528.1 kJ·mol^−1^; for Ca(NO_3_)_2_⋅4H_2_O, this value is −2129 kJ·mol^−1^, and for Mg(NO_3_)_2_⋅6H_2_O, enthalpy is −2610 kJ·mol^−1^. The exothermicity of the oxidation–reduction reaction ranges from 727 to 1527 °C. Depending on the fuel used, the combustion behavior differs according to whether a flame is present [92]. With conventional single-phase SCS, it is difficult to obtain metal particles smaller than 15–20 nm and with a specific surface area exceeding 40–50 m^2^·g^−1^, even when different organic fuels are employed [93]. Nanostructured nickel ferrites were synthesized via SCS using glycine, ascorbic acid, oxalic acid, and malic acid for photocatalytic tests. The results revealed that the morphology strongly depended on the composition of the initial reaction atmosphere, with the formation of aggregates ranging in size from 5 to 40 µm. Samples prepared with oxalic acid and malic acid exhibited the smallest particle sizes and the highest specific surface areas.

The dependence of the O/C ratio on the valence of the reducing fuel plays an important role in the combustion of solutions. The O/C ratio is calculated considering the overall oxidation reaction for each fuel. For example, the complete oxidation of glycine with oxygen is described in Equation (5):

(5) $$C_{2}H_{5}NO_{2}+\left(\frac{9}{4}\right)\;O_{2}\to \left(\frac{5}{2}\right)\;H_{2}O+2CO_{2}+\left(\frac{1}{2}\right)\;N_{2}$$

To calculate the O/C molar ratios, the following Equation (6) is used:

(6) $$\frac{o}{c}=\left(\frac{mol\;O_{\left(fuel\right)}+mol\;O_{{(O}_{2})}}{mol\;C_{\left(fuel\right)}}\right)=2\left(\frac{moi\;O_{{(H}_{2}O)}}{moi\;O_{{(CO}_{2})}}+1\right)$$

where O_(fuel)_ is the mole of oxygen atoms from the fuel, O_(O2)_ is the mole of oxygen atoms from an oxygen molecule, and C_(fuel)_ corresponds to the moles of carbon from the fuel. Note that the stoichiometric coefficients in Equation (5) must be taken into account. Alternatively, O_(H2O)_ and O_(CO2)_ can be considered, which correspond to the moles of oxygen atoms from water and carbon dioxide, respectively [94].

Organic substances synthesized by the solution combustion method for use as fuel for catalysts must have the following properties:

- The fuel must be soluble in water and used to improve solubility;
- The fuel can act as a dispersing or complexing agent for the metal, which forms a new metal-fuel precursor;
- The melting point of the organic fuel should generally be below 250 °C, and its ignition temperature should be below 500 °C;
- The fuel should typically decompose completely, releasing large amounts of gases that improve the textural properties of the catalyst;
- It should be compatible with metal nitrates, i.e., the combustion reaction should be controlled and smooth and should not lead to an explosion;
- It should not produce any residual mass other than the oxide in question;
- It should be readily available or sufficiently easy to synthesize [95,96].

In solution combustion, the fuel is used as a reducing agent to provide the necessary heat, promote increased solubility of oxidants, and prevent ion precipitation during evaporation and removal of water. In solution combustion, fuel performs two important functions: it acts as a chelating agent and a microstructural model. The chelating agent forms stable complexes for the formation of composite materials and microstructural models [97]. When using glycine and urea as fuel, the synthesized powders were observed to have a variety of microstructures and textures [98]. The influence of the oxidizer-to-fuel ratio is described in [99], which presents the synthesis of nanocrystalline Al_2_O_3_ powder by solution combustion. Aluminum nitrate and urea were used as reactants. The influence of stoichiometric nitrate-to-fuel ratios was studied. Compositions in which the fuel-to-oxidizer ratio corresponded to 40%, 80%, and 120% were used in that study. During the experiment, a very high flame was formed with the addition of 120% fuel, which led to an increase in the exothermicity of the reaction. The authors found that urea had the best potential for the production of pure alumina powder. This alumina is widely used for the preparation of stable tiles, faucet washers, seals, electronic substrates, cutting tools, bioceramics (hip joints), armor materials, laboratory glassware, and wearing parts in the textile and paper industries, as well as for the production of monolithic and refractory products.

Studies [21,100] examined the mechanism of SCS in nickel nitrate–glycine systems at varying fuel-to-oxidizer ratios (φ). It was found that the φ ratio and water content significantly influence the combustion behavior, phase composition, crystallite size, lattice parameters, pore structure, and surface area of the resulting catalyst. Combustion temperature and velocity were observed to increase with higher fuel content, reaching 0.39 cm·s^−1^ at φ = 1.0. XRD analyses revealed that at lower φ values (around 1.0), both Ni and NiO phases form, while at slightly higher ratios (φ ≈ 1.25), pure metallic nickel is obtained. These results demonstrate that the fuel-to-oxidizer ratio plays a crucial role in controlling the phase and microstructural characteristics of SCS-derived Ni-based catalysts.

The effect of fuel composition on phase stability, morphology, and crystallite size was studied [101]. Aluminum nitrate and urea were used for synthesis, while starch ((C_6_H_10_O_5_)_n_) or ammonium nitrate (NH_4_NO_3_) was used as a fuel additive. With the addition of ammonium nitrate, well-crystallized α-Al_2_O_3_ powders with particle sizes ranging from 20 to 800 nm were obtained. With the addition of starch, amorphous alumina powder was obtained. However, after heat treatment in a muffle furnace at 1100 °C for 1 h, it transformed into crystallized α-Al_2_O_3_ with a particle size of 30~50 nm.

Using glycine and urea, NiO powder was synthesized via solution combustion. The phase composition of the resulting products was studied using X-ray diffraction, which revealed that the synthesized catalysts formed Ni phases when glycine was used, with a fuel-to-oxidizer equivalence ratio of 1.4. No Ni phase was formed when urea was used as the fuel. The powders synthesized using glycine had a branched, cotton-like structure consisting of soft agglomerates, while the powders synthesized using urea were spherical with hard agglomerates. On average, less residual carbon was found when using glycine as a fuel. The powders synthesized using urea had better sinterability due to their small crystallite size and spherical shape [102].

The glycine fuel method is well suited for the production of nanoscale composites. Some characteristic features of the process can be noted:

- Oxides and their mixtures can be prepared at very low temperatures < 400 °C;
- The products are homogeneous and crystalline;
- They are soft in structure with a high surface area;
- The synthesized materials are highly pure (99.99%);
- The particles are less agglomerated and can be used directly for coating;
- Glycine is a relatively inexpensive substance that can be used to prepare a large number of composite materials [103].

Another approach to SCS is the preparation of catalyst supports (γ-alumina) and mixed fuel (a combination of urea and glycine) [104]. In this research, Al(NO_3_)_3_·9H_2_O reacted with urea, while glycine served as the fuel and was added in excess. The changes in phases, according to XRD, were very interesting: the high-temperature α-Al_2_O_3_ was transformed into a mixture of α-Al_2_O_3_ and γ-Al_2_O_3_. The high-temperature form is very stable at 1200 °C. Two-fuel combustion can enhance catalytic activity, which is one of the advantages of SCS [105].

The results of a study of catalysts based on a Co(NO_3_)_2_ + Μg(NO_3_)_2_ + H_3_BO_3_ + urea/glycine + H_2_O system, obtained by solution combustion, are presented in [106]. The catalysts were synthesized from Co(NO_3_)_2_, Mg(NO_3_)_2_, and H_3_BO_3_. Urea or glycine was used as the fuel reducing agent, and water was added as the solvent. Synthesis was carried out at a preheated furnace temperature of 500 °C. During combustion in SHS mode, the following reactions were presumably observed (Equations (7)–(13)):

Co(NO_3_)_2_·6H_2_O → CoO + H_2_O + NO + NO_2_ (7)

Mg(NO_3_)_2_ + 5CH_4_N_2_O → MgO + N_2_ + CO_2_ (8)

B_2_O_3_ + CoO → CoB_2_O_4_ (9)

2B_2_O_3_ + MgO → MgB_4_O_7_ (10)

Mg + Co_3_O_4_ → Co_3_MgO_4_ (11)

Mg + Co_3_O_4_ → Co + MgO (12)

Mg + Co_3_O_4_ → Co_x_Mg_y_ (13)

Thus, catalysts were obtained by solution combustion based on the system Co(NO_3_)_2_–Mg(NO_3_)_2_–H_3_BO_3_–urea/glycine–H_2_O, and comprehensive studies of their physicochemical properties have been conducted.

Glycine, when used as a reducing agent with nitrate, produces more gases than when using urea. This results in the formation of a more porous structure than that obtained with urea. The more gas is present, the more heat is removed during combustion. This results in a lower combustion temperature, which affects the final catalyst composition. For example, when using urea (with the same initial batch composition), the concentration of cobalt cations in the spinel lattice is higher. Urea produces Co_3_MgO_4_, while glycine produces a greater amount of CoMg_3_O_4_. The activity of the obtained catalysts, 60% Co(NO_3_)_2_ + 40% Mg(NO_3_)_2_ + 22% urea + 37% H_3_BO_3_ and 60% Co(ΝO_3_)_2_ + 40% Mg(NO_3_)_2_ + 22% glycine + 37% H_3_BO_3_, was studied in the process of carbon dioxide reforming of methane in the temperature range of 750–900 °C at GHSV = 860 h^−1^.

During catalyst testing (from the initial charge of 60% Co(NO_3_)_2_ + 40% Mg(NO_3_)_2_ + 22% urea + 37% H_3_BO_3_), the maximum conversion of CH_4_ and CO_2_ was found in the regions of 40.5% and 38.74%, respectively. Compared to this sample, the catalyst from the initial charge of 60% Co(NO_3_)_2_ + 40% Mg(NO_3_)_2_ + 22% glycine + 37% H_3_BO_3_, containing glycine, exhibited higher activity than the catalyst containing urea. CH_4_ conversion on it reached 54.55%, while CO_2_ conversion reached 51.74%. The H_2_ and CO yields reached 54% and 51%, respectively, sat a temperature of 900 °C [106].

**Figure 1:**
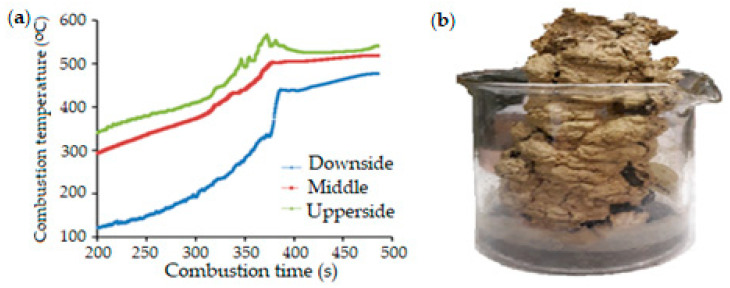
Temperature–time profile of volumetric combustion for Ni-Cr-Al-Mg catalyst (**a**); freshly prepared catalyst obtained via SCS (**b**).

Ni-Cr based catalysts promoted by Mg were synthesized via combustion in a solution with glycine. Figure 1 shows the temperature–time profile of the volumetric combustion of 25% Ni(NO_3_)_2_ + 5% Cr(NO_3_)_3_ + 10% Al(NO_3_)_3_ + 10% Mg(NO_3_)_2_ + 50% glycine catalyst [107].

**Figure 2:**
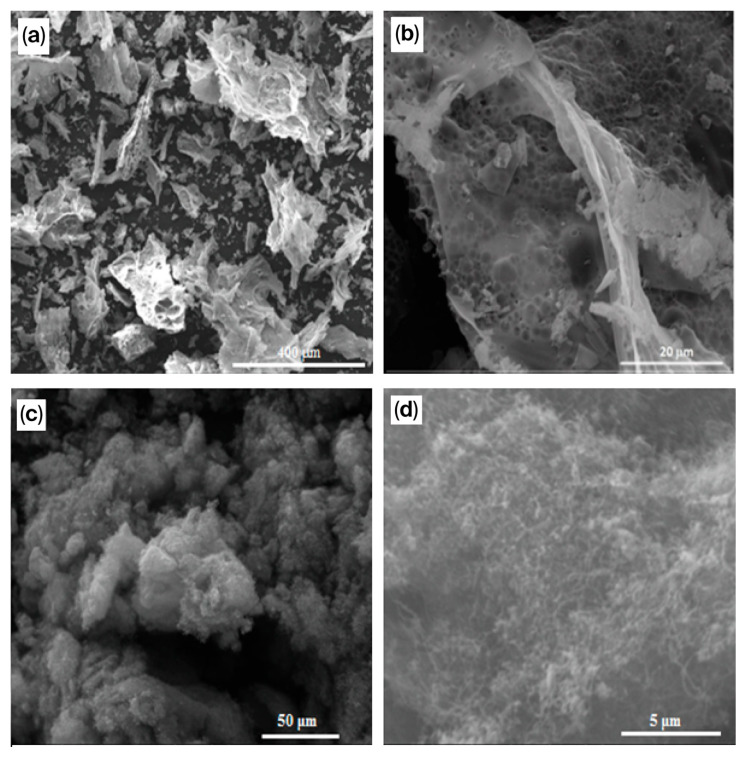
Electron microscopic images obtained with a scanning microscope for 25% Ni + 5% Cr + 10% Mg + 10% Al initial (**a**,**b**) and spent (**c**,**d**) catalysts.

Figure 1 shows the progression of the volumetric combustion process. Water evaporated for up to 300 s, after which a gel formed. During this process, different temperatures were observed in the lower, middle, and upper regions. After 375 s, the temperature increased rapidly, and gas formation was visible in the muffle furnace. This catalyst was studied under the following conditions in dry methane reforming: GHSV = 2500 h^−1^; CH_4_: CO_2_: Ar = 1:1:1. From the results, it is evident that at 800 °C, the conversion of both CH_4_ and CO_2_ begins to increase, and at 900 °C, they reach 99% and 98% conversion, respectively. Figure 2b shows that with increasing temperature, the yields of H_2_ (39%) and CO (60%) increase, as does the H_2_/CO ratio in the range of 1.5–1.7.

It is well known that in solution combustion synthesis, powder morphology, particle size, and surface area are directly related to the amount of gases released during combustion. Gases break up large clusters and create pores between the particles. In fact, clusters disintegrate when high amounts of combustible gas are produced, and under these conditions, more heat is released from the system, inhibiting particle growth. The difference in particle size when using different fuels depends on the number of moles of gaseous products released during combustion.

Figure 2 shows electron micrographs of a 25% Ni(NO_3_)_2_ + 5% Cr(NO_3_)_3_ + 10% Al(NO_3_)_3_ + 10% Mg(NO_3_)_2_ + 50% glycine catalyst prepared by solution combustion.

Figure 2a shows the formation of crystals during catalyst preparation. It should be noted that they are nanosized (Figure 2b). Under magnification, fine bubbles are visible, as are nanopores formed during combustion. Micrographs of the spent catalysts demonstrate a foam-like morphology with a highly macroporous texture characteristic of solids prepared by solution combustion (Figure 2c,d). This catalyst was stable for 72 h due to the presence of Cr and Mg and Al, which served as supports in the MgAl_2_O_4_ spinel.

**Figure 3:**
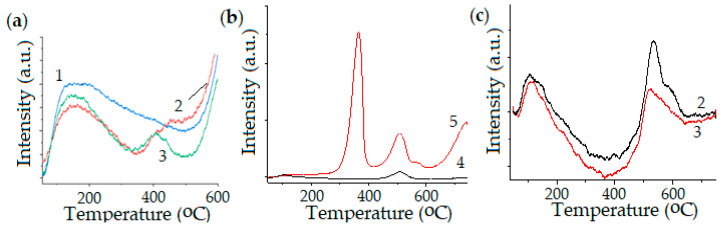
Acidity and basicity of some catalysts prepared by SCS [108]. (**a**) NH_3_ TPD; (**b**,**c**) CO_2_ TPD profiles; 1—15Ni-35Al, 2—15Ni-15Ce-20Al, 3—15Ni-15La-20Al, 4—15Ni-35Ce, 5—15Ni-35La.

The authors of [87] studied iron-promoted catalysts, finding that the lower the iron content, the higher the activity due to the active phase of NiAl_2_O_4_ in 15Ni-5Fe-30Al, since its decomposition into an Al_3_Ni_2_ alloy and Al_2.667_O_4_ occurred during DRM. A small metal particle size, high reducibility, and strong metal–support interactions facilitate high rates of conversion of methane and CO_2_ and a high synthesis gas yield. Higher iron loadings cause the formation of stable and unreduced FeAl_2_O_4_. Metal sintering was also observed for these catalysts. It is worth noting that the addition of a small amount of Fe increased the catalyst’s stability for 20 h. The reaction conditions were as follows: GHSV = 3000 h^−1^; CH_4_: CO_2_:Ar = 1:1:1. The initial CH_4_ conversion was 92%, and CO_2_ conversion was 81%, which remained steady throughout the stream. For the 15Ni-5Fe-30Al catalyst, the Fe/Ni molar ratio was 0.26 [75,89]. SEM images showing the morphological properties of the 15Ni-5Fe-30Al catalyst are presented in Figure 3. In these images, an erratic, flaky structure with dimensions ranging from 60 to 420 μm can be observed on the surface. These particles possess microporosity and display cracks on the catalyst surface. Notably, there were no significant differences in the morphology of the catalysts before and after the reaction.

**Figure 4:**
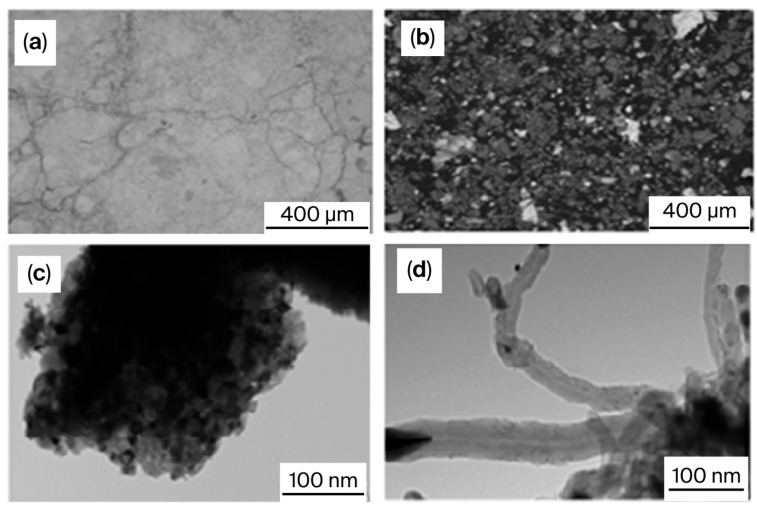
Scanning and transmission electron microscope images of the 15Ni-15Fe-30Al catalyst [89]. (**a**,**b**) SEM images of fresh and spent samples; (**c**,**d**) TEM images of fresh and spent samples.

The TEM results for the 15Ni-15Fe-30Al catalyst show (Figure 4) well-dispersed metal particles. 15Ni-5Fe-30Al had the narrowest nickel particles, explaining its superior performance [75,87]. Carbon nanotubes were consistently present in the spent catalyst.

Interesting results were also obtained for catalysts modified with rare-earth metals, such as Ce and La [109]. These materials were prepared by SCS. 15Ni-15Ce-20Al and 15Ni-15La-20Al were not reduced before the DRM reaction, since CeAlO_3_ and LaAlO_3_ can be transformed into CeO_2_ and La_2_O_3_ and reoxidized into perovskites. Reduction is easier when Al is present. For instance, the simultaneous addition of Ce and La in one catalyst, Ni-Ce-La, decreases the reducibility due to La_2_Ce_2_O_7_. This catalyst was reduced prior to the reaction yet remained less active than its counterparts (Ni-Ce-Al and Ni-La-Al). The stability of the 15Ni-15Ce-20Al catalyst was examined at 850 °C for 20 h, maintaining GHSV of 3000 h^−1^, considering its relatively superior performance. CO_2_ and CH_4_ conversion yielded similar results, remaining stable for 20 h. Simultaneously, the carbon balance was around 75%, and the H_2_/CO ratio ranged from 1.0 to 1.2, demonstrating excellent performance. In addition, the rate of coke accumulation was 1.3 wt.%·h^−1^ for the 15Ni-15Ce-20Al catalyst at 20 h TOS, per CHNS analysis. This rate is attributed to the adsorption of filamentous carbon deposited on acidic and metal sites. The values of conversion over 15Ni-15Ce-20Al were 45% (CH_4_) and 70% (CO_2_). Additionally, NH_3_ and CO_2_ TPD results are presented for 15Ni-35Al, 15Ni-15Ce-20Al, 15Ni-15La-20Al, 15Ni-35Ce, and 15Ni-35La (Figure 4). The highest level of acidity was observed for the 15Ni-35Al catalyst, as it contained the largest amount of Al. In comparison, the 15Ni-15La-20Al catalyst displayed higher acidity than 15Ni-15Ce-20Al, which can be attributed to its marginally higher aluminum content. Each of the three catalysts possessed a higher number of robust acid sites, specifically those with NH_3_ desorption peaks near 600 °C.

**Figure 5:**
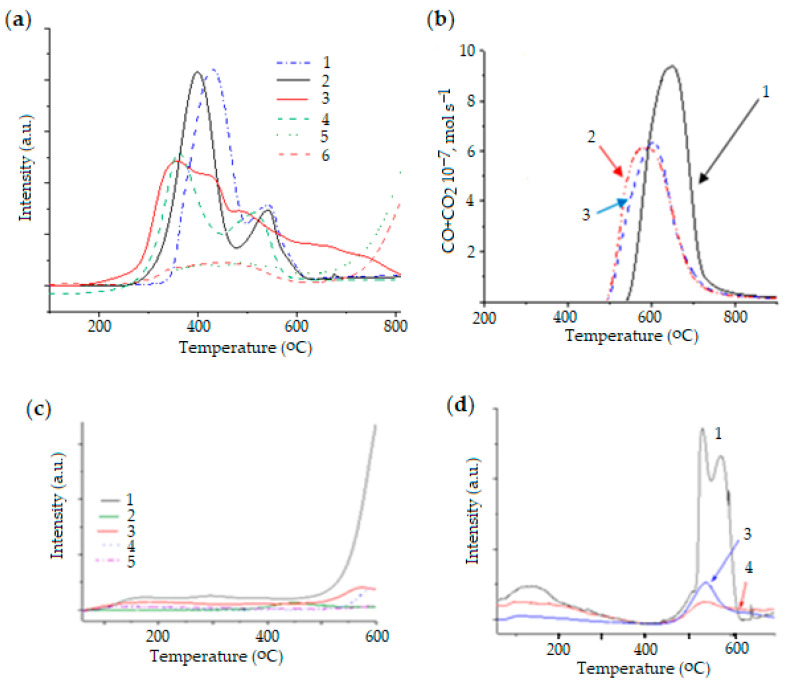
Reducibility, oxidation, acidity, and basicity of Ni-Mn, Ni-Mg based catalysts [64,111]. (**a**) H_2_-TPR: 1—15Ni-35Mg, 2—15Ni-35Mn, 3—15Ni-15Mn-20Al, 4—15Ni-15Mn-20Mg, 5—15Ni-15Mg-20Al, 6—15Ni-35Al; (**b**) O_2_-TPO: 1—15Ni-15Mn-20Mg, 2—15Ni-15Mn-20Al, 3—15Ni-15Mg-20Al; (**c**) NH_3_-TPD, (**d**) CO_2_-TPD profiles; (**c**,**d**): 1—15Ni-35Mg, 2—15Ni-35Mn, 3—15Ni-15Mn-20Al, 4—15Ni-15Mg-20Al, 5—15Ni-35Al.

Ni-Mn-Al, Ni-Mn-Mg, and Ni-Mg-Al catalysts were synthesized by solution combustion [64,110]. The addition of Mn was interesting because there are few publications on the use of this catalyst, which is also applicable in DRM. However, Ni-Mn-Al is more acidic than Ni-Mg-Al, which improves methane decomposition and leads to more coke formation. According to the XRD, TPR, and TPD results, the Ni-Mn-Mg had Ni-Mn and/or Ni-Mg solid solutions demonstrated enhanced acidic sides and higher amounts of carbon, even exceeding those of Ni-Mn-Al. The temperature-programmed oxidation (TPO) results indicate that carbon release was 622 °C for Ni-Mn-Mg and 559 °C for Ni-Mn-Al, correlating with temperature-programmed desorption (TPD), temperature-programmed reduction (TPR), and XRD results (Figure 5).

For the Ni-Mg-Al catalyst, the absence of valent nickel enhanced catalytic activity due to strong metal–support interactions, low carbon, and intense basicity of the MgAl_2_O_4_. In a stability test, this catalytic system was proven stable for 200 h under harsh conditions (800 °C).

Thus, the fresh 15Ni-35Mn and 15Ni-35Mg catalysts contained manganese oxide and magnesia phases, while the spent samples contained metallic nickel. The addition of aluminum to catalysts strengthened the interaction between the metal and the carrier, which improved their activity in the reaction. The catalysts are basic, resulting in insignificant coke format, which was confirmed by the CO_2_ TPD and CHNS methods.

**Figure 6:**
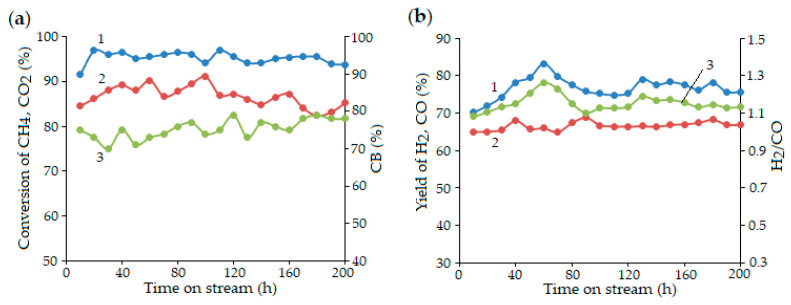
Behavior of 15Ni-15Mg-20Al in a long-term stability examination in DRM [64]. (**a**): 1—conversion of CH_4_, 2—conversion of CO_2_, 3—carbon balance, (**b**): 1—yield of H_2_, 2—yield of CO, 3—H_2_/CO; GHSV = 3000 h^−1^, T = 800 °C.

As shown in Figure 6, 15Ni-15Mg-20Al was tested in DRM for 200 h under the following conditions: GHSV = 3000 h^−1^; T = 850 °C [64].

The conversion of methane and CO_2_ stabilized after 20 h, showing only minor fluctuations within ranges of 94–96% and 84–90%, respectively, throughout most of the experiment. After 40 h, CO_2_ conversion slightly increased to approximately 90%. The CO yield remained nearly constant during the entire 200-hour test. Synthesis gas was generated with an H_2_/CO ratio of 1.2, while the overall carbon balance (CB) ranged between 69% and 80%, as shown in Figure 6. In the final 24 h, condensed water vapor was detected, indicating a possible reverse water–gas shift (RWGS) reaction; however, CO_2_ conversion still remained below that of CH_4_. Interestingly, for the 15Ni-15Mg-20Al catalyst, the coking rate determined by the CHNS analysis decreased with increasing time on stream [64].

**Figure 7:**
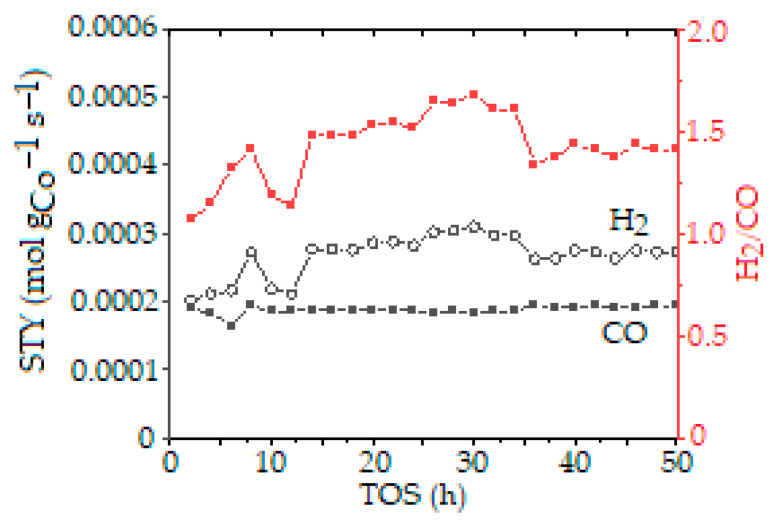
Behavior of CoLaAl catalyst in the 50 h TOS stability examination in DRM [112]. 800 °C; GHSV = 3000 h^−1^.

The Co-based catalyst promoted by lanthanum was also examined to compare its catalytic activity and stability for 50 h (Figure 7) with that of Ni-Mg-Al [112]. The CoLaAl catalyst, synthesized via SCS, showed small metal particle sizes (19–25 nm) and contained a perovskite-type mixed oxide phase, LaCoxAl_1−x_O_3_, which was isomorphic with LaAlO_3_. XRD analysis of the spent CoLaAl catalyst showed no carbon deposits, while other catalysts contained graphite-type carbon. Since CoLaAl exhibited the least coke formation in comparison with CoAl and CoCeAl, it was examined in a long-term test (50 h) and demonstrated stable CH_4_ and CO conversions, with an H_2_/CO ratio fluctuating between 1.1 and 1.7. However, the perovskite phase, LaCoxAl_1−x_O_3_, was found to decompose after 20 h on stream, indicating limited long-term structural stability despite its excellent initial performance and coking resistance.

Thus, the mechanism of SCS is governed primarily by the properties of the organic fuel, the fuel-to-oxidizer ratio, and the water content in the precursor solution. These criteria define the flame temperature, thermodynamics, microstructural evolution of the resulting materials, and gas evolution. Variations in fuel composition or the F/O ratio obviously alter phase formation, porosity, crystallite size, and dispersion. For instance, as was mentioned above, use of fuel-rich compositions led to combustion at higher temperatures and faster self-propagation, while fuel-deficient systems were susceptible to forming mixed phases. The ability of SCS-derived materials to form spinels, perovskites, solid solutions, and highly dispersed metallic phases at relatively low pre-ignition temperatures (<400–600 °C) underscores the method’s versatility. SCS catalysts are used in catalytic reactions such as DRM due to their high dispersion, thermal resistance, superb activity, and resistance to sintering and coking, making SCS a powerful method for designing next-generation catalysts for dry reforming and related high-temperature reactions.

### 2.6. Catalyst Deactivation and Methods of Decrease

#### 2.6.1. Catalyst Deactivation During Dry Reforming of Methane

Deposition of coke on the catalyst surface is anticipated by elevated temperatures sufficient to break the C-H bonds in CH_4_ [108]. The main problem with nickel-supported catalysts is their acceleration of carbon generation reactions, which decrease catalytic activity [111]. Amorphous (C_α_), filamentous (C_β_), and graphitic (C_γ_) carbon occurs in the treatment of organic compounds and methane-reforming processes [108,113]. Amorphous coke in the form of carbide can be hydrogenated at low temperatures. Filamentous carbon is usually formed at temperatures above 500 °C, occurring in most processes, while graphite formation is generally observed during treatment of heavier hydrocarbons at ca. 650 °C. At this temperature, ethylene is obtained by the cracking of long-chain hydrocarbons, which can be a result of the deposition of graphite on the catalyst. Graphite completely encapsulates the catalyst particles, causing deactivation. If polymerized by hydrocarbons during treatment, amorphous coke can be transformed into pyrolytic coke and contains thin hydrocarbon films.

Using DFT and Kinetic Monte Carlo simulations, the authors of [114,115] showed that carbon species from C1 to C9 favor chain configurations as the most stable form, while C10 and C11 prefer ring structures. Additionally, the interaction between C1 and C2 species promotes the growth of long carbon chains, leading to carbon deposition. XRD and DTA analyses can identify graphitic coke, according to [116].

Nevertheless, a unique explanation for the generation of carbon species in a catalyst was not provided. Strong filaments were created by coke diffusion, which occurred during decomposition reactions via the nickel particle on the unexposed side. The generation of filaments grows a nickel particle, which pushes the nickel particle away from the catalyst structure. The growth of filamentous carbon is the result of the following mechanisms:

- Hydrocarbon or carbon monoxide adsorption on the surface;
- Dissociation of carbon monoxide or hydrocarbons to generate adsorbed carbon. Metal is formed at the tips of carbon filaments in the rear interface, where coke diffusion and dissolution occurred [108].

As the metal particles become larger, the strength of the carbon fibers increases, and they push out fragments of the active metal particle. These fibers can destroy the pores of particles. Filamentous carbon can also cause breakage of the catalyst pellets. This can have catastrophic consequences for the reactor, ranging the reduction of activity to blockage and damage to the reactor in the case of steam reforming by hot spots [108].

As a result, carbon growth depends on a combination of temperature and concentration gradient. While carrying out mixed steam and carbon dioxide reforming, even low amounts of CO_2_ can have a drastic effect on carbon generation. An increase in CO_2_ concentration in the feedstock at temperatures below 727 °C increases the steam/carbon ratio to maximize carbon accumulation prevention. Nevertheless, at temperatures higher than 727 °C, a decrease in steam/CO_2_ supports coke formation.

Small metal particles, such as those less than 8 nm, can diminish carbon accumulation [117], since metal–support interactions improve metal nanoparticles and do not allow for carbon diffusion on the metal surface. Proper operating conditions and high catalyst dispersion can prevent catalyst deactivation due to coke formation [108]. In addition, a NiAl_2_O_4_ and MgAl_2_O_4_ spinel phase forms in the Ni/Al_2_O_3_ and NiMg/Al_2_O_3_ systems upon calcination at high temperatures [117,118]. Although the nickel aluminate phase is not active in DRM, it may have an inert, stabilizing, or deactivating effect on the Ni/Al_2_O_3_ system. It was also reported that the formation of a surface spinel phase can effectively reduce coke deposition [118]. Mg addition offers basic benefits, in addition to resistance to carbon [119]. For instance, in [119], a Ni/MgO catalyst was investigated at temperatures above 500 °C. Coke is highly soluble in metal, resulting in the encapsulation of carbon filaments in Ni particles sized in the range of 5–20 nm. Otherwise, large nickel particles facilitate the development of carbon deposits, plugging the pores of the support and blocking the active centers. Ni/MgO and solid solutions supported on alumina are good catalysts because they are stable and highly resistant to carbon formation.

#### 2.6.2. Methods to Decrease Carbon Formation

Essentially, coke influences catalyst effectiveness via two primary mechanisms: either by coating active sites, thereby decreasing the reactant’s access to active sites and causing poisoning, or by obstructing pores. The specific type of coke produced depends on the catalyst’s composition and the process parameters. As a result, regeneration approaches differ depending on the catalytic procedure [120]. Coke can be reduced via oxidation with oxygen and ozone, CO_2_ and steam gasification, pyrolysis under inert gas, and hydrocracking with H_2_ and alkanes.

Catalyst deactivation due to coke buildup is generally reversible. Moreover, coke can be readily burnt by oxygen at 500 °C. Therefore, catalysts are either partially or completely decoked at 450 °C using O_2_. Coke deposition was estimated using TPO, which revealed that optimal coke combustion occurred at 500 °C [121].

Another challenge arises from the fact that the residual coke has the potential to shift from aliphatic to aromatic as it undergoes oxidation. This change adds to the complexity of the regeneration process. For example, ZSM-5 catalysts with coke deposits can be rejuvenated by using ozone at lower temperatures. To align with the principles of “green carbon science”, the integration of catalyst regeneration via the gasification of carbon using either H_2_O or CO_2_ was explored [120]. These procedures are designed to generate synthesis gas as the primary output during the regeneration phase, diverging from the usual production of CO_2_.

Oxidation of coke is exothermal process that results in the production of H_2_O or oxides of carbon as the primary components of the flue gas. For a coke content of 1.5% on HY, carbon oxidation initiates at 250 °C, resulting in the removal of ca. 5%. Nevertheless, the majority of carbon is effectively eliminated at 450 °C, and this trend persists at 500 °C, resulting in the removal of approximately 70%. If the coke content is higher, coke oxidation only initiates at 350 °C, with the most significant portion of carbon (ranging from 50% to 60%) being removed at 450 °C. A noticeable pattern emerges: the greater the coke content, the lower the proportion of carbon that necessitates oxidation at 500 °C for its removal. The authors of [122] also discussed the type of coke in relation to the amount. The higher the carbon content on the catalyst surface is, the more aromatic rings are present, indicating its aromatic nature.

Moreover, it was determined [122] that regardless of coke or catalyst properties, ignition of the combustion process consistently commences with the elimination of hydrogen. This leads to the formation of water and oxygenated intermediate compounds. These intermediates either break down into CO and CO_2_ or undergo complete oxidation to form CO_2_ and H_2_O. This observation was validated by identifying the evolution of H_2_O and carbon oxides during a gradual temperature ramp in the presence of oxygen (TPO). Temperatures below 300 °C yielded water in a notable quantity, whereas temperatures surpassing 500 °C only yielded CO_2_ and CO.

However, the oxidation of coke is extremely damaging, and the heat produced could cause irreversible damage to the catalyst. This includes the creation of areas with elevated temperatures (hot spots), localized intense temperature variations, and the overall degradation of the catalyst. Eliminating coke positioned close to the metal is relatively easier than eliminating coke on the support matrix, which demands longer regeneration periods and elevated temperatures.

To counteract the potential for thermal damage while restoring coke, maintaining control over the temperature is critical. One effective strategy is to alter the catalyst metal component. This can aid in the management of the regeneration process [120].

Since ozone possesses strong oxidizing features, a mixture of ozone and oxygen (O_3_/O_2_ mole ratio = 0.04) can remove carbon from catalysts at 50–200 °C during regeneration [123]. The performance of a H-ZSM-5 catalyst deactivated after methanol conversion was restored using this regenerating mixture at 150 °C for 90 min. In comparison to reactivation with oxygen, the use of ozone for regeneration resulted in a slightly longer catalyst lifespan but decreased the initial methane yield. Restoration by ozone at low temperatures proves to be an effective method that carries minimal risk of metal sintering, dealumination, or hydrothermal degradation. The main disadvantages of regeneration using ozone lie in its rapid dissociation, limitation regarding pore diffusion, and stringent regulations on O_3_ emissions, with a maximum permissible limit of 75 parts per billion to prevent atmospheric damage.

An alternative method of catalyst regeneration involves employing CO_2_ gasification, since carbon dioxide can serve as a gentle oxidizing agent during its reaction with carbon [124]. The reverse Boudouard reaction preferred in this case and is shown below (Equation (14)):

C + CO_2_ → 2CO + 172 kJ·mol^−1^ (14)

The regeneration of coke-fouled catalysts through CO_2_ gasification offers the advantage of reducing CO_2_ to CO, thus contributing to a more favorable carbon footprint. However, CO_2_ gasification is an extremely energy-consuming reaction that typically occurs at temperatures exceeding 700 °C. These high temperatures can potentially lead to catalyst structural damage and/or metal sintering. Hence, the requirement for elevated reaction temperatures and the limited reactivity of CO_2_ are the primary constraints for catalyst regeneration through CO_2_ gasification. However, this approach offers some advantages over steam gasification. Unlike steam, CO_2_ is already in a gaseous state and does not require vaporization prior to the gasification process. Furthermore, steam potentially reacts with the Al-O bond in the catalyst at elevated temperatures, which can result in the structural breakdown of the catalyst. Notably, CO_2_ gasification does not have a detrimental effect on the catalyst structure.

Higher temperatures positively influenced the gasification rate [124]. However, as the coke conversion surpassed 50%, the rate notably decreased. The removal of coke deposits occurred in two stages: initially, the coke located on the catalyst surface was eliminated. Thereafter, the coke located within the pores was removed. In this scenario, as the reaction progresses, Knudsen diffusion gradually becomes the dominant factor, resulting in a decline in the gasification rate.

CO_2_ gasification is applied in regenerating Ni catalysts for reformation of hydrocarbons with steam. The temperature of coke combustion was above 327 °C. Furthermore, the long-term performance of the regenerated catalysts declined slowly as the number of reaction–regeneration cycles increased. A study revealed that CO_2_ gasification could partially remove coke deposits on deactivated Ni/Y_2_O_3_-La_2_O_3_-ZrO_2_ catalysts utilized in steam reforming of ethanol, guided by incomplete restoration of catalyst activity [125]. In the case of Ni/12Zr29Y13La, among binary oxide supports such as Ni/35Zr14La and Ni/36Zr14Y, substantial production of H_2_ and minimal presence of disordered carbon residues at the lowest coke combustion temperature of 327 °C were observed.

Three consecutive cycles and subsequent regeneration were executed. The restoration phase involved the examination of two distinct gasifying media: CO_2_ (21% CO_2_/N_2_) and air. The primary objective was to assess the catalyst’s capacity for restoration across diverse atmospheric conditions at 700 °C [126]. The catalyst demonstrated consistent performance for both atmospheres with minor deactivation. Irreversible loss of activity between cycles occurred due to the development of core–shell carbon structures and nickel particle agglomeration during the reaction. However, following agglomeration, the nickel particles exhibited a spherical morphology and small sizes (ca. 10–50 nm), which hindered additional carbon accumulation. Despite the modest decline in reactant conversion, syngas selectivity remained stable, implying that deactivation had no impact on selectivity. These outcomes underscore the potential for convenient in situ Ni/Ca-HA1-S regeneration under varying atmospheres, rendering it a strong contender for DRM. Additional experimentation is necessary to fine-tune the regeneration process and diminish the deposits of core–shell carbon. In [127], only 20 vol.% O_2_/80 vol.% Ar was used as a regeneration agent after DRM. Regeneration influenced catalytic activity but did not decrease it.

The utilization of steam in regeneration offers the advantage of reducing the accumulation of CO_2_ while generating syngas consisting of H_2_ and CO. However, at high temperatures, it can result in structural damage to the catalyst and stable deactivation. Steam gasification can be represented by Equation (15):

C + H_2_O (g) → CO + H_2_ (15)

Measuring the H/C ratio in the remaining carbon in the spent SAPO-34 catalyst can be challenging. However, a combination of TGA and GC-MS techniques can be employed to examine the qualitative shift in the H/C ratio in the residual coke. During TGA analysis, coke portions with hydrogen deficiency, indicating lower H/C ratios, tend to undergo combustion at higher temperatures (TG,_max_) [128].

Coke gasification (including bituminous coal chars, commercial coke, and lignite) using H_2_O is approximately 2–5 times faster compared to gasification with CO_2_ [129]. However, steam gasification of coke species with a less interactive or graphitic nature demands elevated temperatures, typically within the range of 700–900 °C. The combination of elevated temperatures and the hydrothermal instability of the catalyst restricts the applicability of steam gasification for removing coke in industrial processes.

Coke elimination can also be achieved through non-oxidative methods, such as hydrocracking using hydrogen [130]. After the USHY zeolite was coked during the transformation of m-xylene at 350 °C under N_2_ flow, it underwent regeneration with H_2_ at 500 °C for 15 h [130]. The coke content was equal to 2.7%. The regenerated catalyst almost entirely reverted to its original catalytic activity, and the deactivation pattern with respect to time on stream closely resembles that of the fresh sample. Nevertheless, coke was not fully removed from the catalyst after H_2_ regeneration. The coke content was reduced from an initial 2.7% to 1.74% following H_2_ treatment. The low coke content could account for the comparable catalytic performance of the regenerated and fresh zeolite. The hydrogenation of coke is described in Equation (16).

Pt-based catalysts are commonly used in propane dehydrogenation, but they tend to deactivate rapidly due to coke accumulation. Sun et al. [131] compared various gases (H_2_, N_2_, and air) for regenerating coked Pt-based catalysts and found that catalysts regenerated by reduction with H_2_ exhibited the highest level of stability. The H_2_ regeneration process not only effectively removed the more resistant coke deposits but also resulted in an increase in the H/C ratio in the remaining coke (Equation (16)).

C (s) + 2H_2_ (g) → CH_4_ (g)–75 kJ·mol^−1^ (16)

Overall, in industrial processes, coke deposits on catalysts are commonly removed through oxidation, gasification, or hydrogenation. Among these methods, air regeneration is often preferred due to its ability to operate at moderate temperatures. Although ozone and oxynitride have the potential to lower the regeneration temperature, their use is restricted due to their harmful nature and strict requirements for emission control. The gasification of coke using CO_2_ or steam offers the advantage of transforming low-value coke into important syngas with a simultaneous decline in CO_2_ emissions. Nevertheless, coke gasification is an energy-intensive process that requires high temperatures, posing a risk of catalyst structural damage. Hydrogenation at higher pressures or temperatures is good approach to coke removal.

## 3. Conclusions and Future Perspectives

This review provides an overview and introduction to the history of solution combustion synthesis. The Ni- and Co-containing catalysts described in this review were selected for their carbon resistance, high catalytic performance, and good stability during long-term DRM. These properties were achieved by the selection of the appropriate fuel (urea or glycine); promoters, based on the transition (Cr, Fe, and Mn); and alkaline-earth (Mg) and rare-earth (La and Ce) metals. Conclusions based on an analysis of the information presented in this review are provided below. New catalyst systems have been developed to obtain active, efficient, and thermally stable catalysts prepared using a modern solution combustion method for producing synthesis gas from methane and CO_2_. The presence of simple and mixed oxides, metal aluminates, and spinel-type structures in the Co(NO_3_)_2_–Mg(NO_3_)_2_–H_3_BO_3_–urea/glycine catalysts were established. Their presence promotes the active operation of the catalysts for the oxidative conversion of methane. The addition of urea to the catalysts results in a higher concentration of cobalt cations in the spinel lattice. In this case, Co_3_MgO_4_ is formed, while when glycine is used, the formation of CoMg_3_O_4_ is preferred. The stability of Ni–Cr–Mg–Al systems synthesized by solution combustion was established for the first time. It was found that the catalyst did not lose its activity for 72 h due to the presence of the Cr and MgAl_2_O_4_ spinel. The properties of the catalysts were studied using XRD, CO_2_-TPD, TGA, TPO, and CHNS methods, which confirmed the role of metallic nickel and metal aluminates in activation, stabilization, inhibition of carbon deposition, and sintering of catalysts in long-term tests. The activity and stability of the Fe-, Mn-, Mg-, Ce-, and La-promoted catalysts in catalytic DRM were described, and the interrelations of their catalytic and physicochemical properties were discussed. In addition to establishing the forms of active elements—alloys, metals, and aluminates—the presence of acid and basic sites in catalysts was shown to play a crucial role in catalysis, depending on the composition of the catalyst and the processing conditions. The substitution of magnesium in the Ni-Mn-Mg catalyst with Al increases its activity, reduces acidity, and leads to the formation of a large amount of coke.

DRM continues to attract attention as a sustainable route for syngas production and CO_2_ utilization. Despite remarkable progress in catalyst design and process optimization, several obstacles still hinder its practical implementation. Catalyst deactivation due to carbon deposition and sintering, high operating temperatures, and the limited long-term stability of active phases remain key obstacles. Future research should therefore focus on developing catalysts with enhanced resistance to coking and thermal degradation. In particular, bimetallic and trimetallic systems, perovskite- and spinel-type oxides, and high-entropy materials show great potential for achieving stable performance under harsh DRM conditions.

The integration of advanced preparation strategies—such as solution combustion synthesis, atomic layer deposition, and microwave-assisted methods—can offer better control over particle size, metal dispersion, and metal–support interactions. Moreover, combining DRM with renewable energy sources or coupling it with other reforming reactions (steam or partial oxidation) could improve overall energy efficiency and tailor the H_2_/CO ratio for downstream applications. The use of in situ and operando characterization techniques will be essential for revealing active sites, understanding reaction mechanisms, and guiding rational catalyst design.

Future developments should also consider process intensification through novel reactor configurations, such as membrane or plasma-assisted systems, and perform comprehensive techno-economic and life-cycle analyses to evaluate industrial viability. With continued innovation in catalyst engineering, reaction integration, and process design, DRM has the potential to become a cornerstone technology for carbon-neutral syngas production in the coming decades.

## Figures and Tables

**Table 1:** Comparison of catalysts prepared by different synthesis methods.

Entry	Catalyst	Synthesis Method and Conditions	DRM Conditions	Initial Conversion of CH_4_/CO_2_ (%) H_2_/CO	Final Conversion of CH_4_/CO_2_ (%) H_2_/CO	Surface Area (m^2^·g^−1^) d_fresh_ (nm)	TOS (h)	Reference
1	Ni-La_2_O_3_	SCS; glycine as fuel; calcination at 550 °C for 2 h	CH_4_/CO_2_ = 1; T = 700 °C; 30 l·g_cat_^−1^·h^−1^	7/20 0.1	60/70 0.75	37.3 10.3	100	[55]
2	Mg-Ni-La_2_O_3_	SCS; glycine as fuel; calcination at 550 °C for 2 h	CH_4_/CO_2_ = 1; T = 700 °C; 30 l·g_cat_^−1^·h^−1^	19/31 0.5	80/90 0.9	54.5 14.8	100	[55]
3	10NiOMgO	PACS; glycine as fuel; pre-ignition at 200 °C	CH_4_/CO_2_ = 1; T = 600 °C; 72 l·g_cat_^−1^·h^−1^	83/95 n.a.	79/94 n.a	163 10	25	[56]
4	15 wt.% Ni/Mg-Al	Sol–gel; calcination at 650 °C for 5 h	CH_4_/CO_2_ = 1.4; T = 800 °C; 34 l·g_cat_^−1^·h^−1^	95/97 n.a.	95/97 n.a.	184 5	8	[57]
5	Ni-Al_2_O_3_-CeO_2_	Sequential impregnation; calcination at 500 °C for 2 h	CH_4_/CO_2_ = 1; T = 850 °C; 24 l·g_cat_^−1^·h^−1^	84/87 0.6	75/80 0.6	65 44	24	[44]
6	Ni-CeO_2_/SiO_2_(CSC)	Colloidal solution combustion; glycine as fuel; calcination at 600 °C for 4 h	CH_4_/CO_2_ = 1; P = 0.1 MPa; T = 700 °C; 120 l·g_cat_^−1^·h^−1^	75/85 1.0	77/85 1.0	63.5 n.a.	20	[63]
7	15Ni-15Mg-20Al	SCS; urea as fuel; pre-ignition at 500 °C	850 °C; CH_4_/CO_2_ = 1; 3 l·g_cat_^−1^·h^−1^	90/84 1.1	92/81 1.2	10 16	200	[64]
8	25Ni-MgAl_2_O_4_	Co-precipitation; calcination at 550 °C for 4 h	750 °C; CH_4_:CO_2_:Ar = 1:1:8; GHSV = 20,000 h^−1^	90/92 1.0	90/92 1.0	115 n.a.	24	[65]
9	12.5 wt.% Ni/CeO_2_	One-pot method; calcination at 400 °C for 4 h	800 °C; CH_4_:CO_2_:Ar = 1:1:1; GHSV = 24 l·g_cat_^−1^·h^−1^	90/100 0.9	90/95 0.9	66.9 n.a.	50	[66]
10	5Ni/SiO_2_-S1	Seed-directed synthesis; 150 °C for 24 h in autoclave	700 °C; CH_4_:CO_2_:Ar = 1:1:2; GHSV = 750 l·g_cat_^−1^·h^−1^	74/85 0.8	71/80 0.8	401 2.9	28	[67]
11	5Ni/La_2_O_3_-LOC	wet impregnation; calcination at 600 °C for 2 h	700 °C; CH_4_:CO_2_:N_2_ = 15:15:70; GHSV = 60 l·g_cat_^−1^·h^−1^	74/82 0.9	70/75 0.9	23 13.8	50	[58]
12	10Ni3Mn4Mg/Al_2_O_3_	Impregnation; calcination at 500 °C for 4 h	700 °C; CH_4_:CO_2_ = 1:1; GHSV = 12 l·g_cat_^−1^·h^−1^	65/70 n.a.	65/70 n.a.	166 n.a.	20	[68]
13	Fe_5%_Ni_5%_Al_2_O_3_	Evaporation-induced self-assembly; calcination at 600 °C for 5 h	700 °C; CH_4_:CO_2_:Ar = 1.8:1:14; GHSV = 36 l·g_cat_^−1^·h^−1^	60/90 1.0	50/90 1.0	198 n.a.	13	[69]
14	10Ni-5Co-0.25Ru/MgO-Al_2_O_3_	Two-solvent impregnation; calcination at 750 °C for 2 h	800 °C; CH_4_:CO_2_ = 1:1; GHSV = 1.1·10^−2^ l·g_cat_^−1^·h^−1^	100/94 n.a.	93/93 n.a.	242 n.a.	24	[70]
15	5% Ni- 5% Co/θ-Al_2_O_3_	Wet impregnation; calcination at 500 °C for 2 h	700 °C; CH_4_:CO_2_ = 1:1; GHSV = 4.3·10^3^ l·g_cat_^−1^·h^−1^	75/82 1.3	49/52 1.3	76 22	12	[71]
16	Pt/Ce	Wet impregnation; calcination at 500 °C for 2 h	650 °C; CH_4_:CO_2_:N_2_ = 1:1; GHSV = 36.7 l·g_cat_^−1^·h^−1^	43/40 0.6	49/45 0.6	67 3.3	24	[72]
17	1.0-Pt-12Ni/Mg-Al	Impregnation; calcination at 600 °C for 6 h	700 °C; CH_4_:CO_2_:Ar = 2:2:1; GHSV = 180 l·g_cat_^−1^·h^−1^	n.a./62 n.a.	n.a./58 n.a.	8.8 0.4	30	[73]
18	NiAl_2:1_	Combustion; urea as fuel; pre-ignition at 600 °C; calcination at 700 °C for 3 h	800 °C; CH_4_:CO_2_:He = 1:1:8; GHSV = 240 l·g_cat_^−1^·h^−1^	98/n.a. 1.1	99/n.a. 1.1	<10 n.a.	50	[74]
19	0.5Ni-Fe-Al	“one-pot” evaporation-induced self-assembly; calcination at 750 °C for 5 h	700 °C CH_4_:CO_2_ = 1:1; GHSV = 24 l·g^−1^·h^−1^	60/68 0.9	56/61 0.8	86 7	7	[75]
20	Ni-Mg/Al_2_O_3_ (MNA-2.0)	Co-precipitation; calcination at 850 °C for 6 h	850 °C CH_4_:CO_2_:N_2_ = 10:20:20 sccm.;	90/98 1.4	90/98 1.4	100 13.1	200	[59]
21	Ni-Fe/Mg(Al)O	Co-precipitation; calcination at 800 °C for 5 h	CH_4_:CO_2_:N_2_ = 1:1:2; 500–800 °C; GHSV = 60 l·g^−1^·h^−1^	90/99 1.2	n.a.	153 14.5	200	[60]
22	Ni-La/Al_2_O_3_	Wet impregnation; calcination at 700 °C for 3 h	CH_4_:CO_2_:N_2_ = 1:1:0.3; 700 °C; GHSV = 42 l·g^−1^·h^−1^	64/79 1.0	63/78 1.0	162 14	8	[76]
23	LaNi_0.9_Mg_0.1_AlO_3-δ_	Auto-combustion; calcination at 700 °C for 6–10 h	800 °C; CH_4_:CO_2_ = 1:1; GHSV = 600 l·g_cat_^−1^·h^−1^	0/0 0	56/67 0.47	10 n.a.	15	[77]
24	LaNi_0.95_Rh_0.05_O_3_	Co-precipitation; calcination at 750 °C for 5 h	550 °C; CH_4_:CO_2_:Ar = 1:1:8; GHSV = 20,000 h^−1^	29/35 0.9	45/45 0.9	8 15	25	[78]
25	LaNi_0.9_Ru_0.1_O_3_	Thermal decomposition of citrate precursors; calcination at 1000 °C for 4 h	750 °C; CH_4_:CO_2_ = 1:1; GHSV = 72 l·g_cat_^−1^·h^−1^	10/10 0.1	85/87 0.7	1.9 n.a.	14	[61]
26	La_0.8_Sr_0.2_Ni_0.8_Fe_0.2_O_3_	Sol–gel; calcination at 700 °C for 5 h	700 °C; CH_4_:CO_2_:N_2_ = 1:1:1	41/59 n.a.	81/79 n.a.	13.9 16	24	[62]
27	SrTi_0.85_Ru_0.15_O_3_	Sol–gel; calcination at 750 °C	900 °C; CH_4_:CO_2_:N_2_ = 1:1:1; GHSV = 28.8 h^−1^	93/96 1.0	93/96 1.0	17 n.a.	100	[79]
28	CaZr_0.8_Ni_0.2_O_3-δ_	Sol–gel; calcination at 750 °C for 6 h	800 °C; CH_4_:CO_2_:N_2_ = 1:1:1; GHSV = 28.8·h^−1^	95/96 1.0	95/96 1.0	13.2 n.a.	500	[80]
29	LaNiAl	Co-precipitation; calcination at 850 °C for 4 h	650 °C; CH_4_:CO_2_:N_2_ = 1:1:8; GHSV = 90 l·g_cat_^−1^·h^−1^	50/50 0.9	50/50 0.9	72 18	24	[81]
30	Ni-Co(0.15)	Co-precipitation; calcination at 850 °C for 4 h	650 °C, CH_4_:CO_2_:N_2_ = 1:1:8, GHSV = 90 l·g_cat_^−1^·h^−1^	89/n.a. 1.0	85/n.a. 1.0	80 20	200	[82]

## Data Availability

The data presented in this study are available upon request from the corresponding author.

## Abbreviations

The following abbreviations are used in this manuscript:

CB	Carbon Balance
CHNS	Carbon, Hydrogen, Nitrogen, and Sulfur Analysis
CO_2_-TPD	Temperature-Programmed Desorption of Carbon Dioxide
CCS	Colloidal Combustion Synthesis
DFT	Density Functional Theory
DRM	Dry Reforming of Methane
GC-MS	Gas Chromatography–Mass Spectrometry Analysis
GHSV	Gas Hourly Space Velocity
BET	Brunauer–Emmett–Teller Method
MSI	Metal-Support Interaction
NH_3_-TPD	Temperature-Programmed Desorption of Ammonia
PACS	Paper Assisted Combustion Synthesis
POM	Partial Oxidation of Methane
RWGS	Reverse Water–Gas Shift Reaction
SCS	Solution Combustion Synthesis
SEM	Scanning Electron Microscopy
SHS	Self-Propagation High Temperature Synthesis
SRM	Steam Reforming of Methane
STY	Space Time Yield
TEM	Transmission Electron Microscopy
TGA	Thermogravimetric analysis
TOS	Time of Stream
TPO	Temperature-Programmed Oxidation
TPR	Temperature-Programmed Reduction
